# Evidence for early endothelial dysfunction associated with the *ALDH2* rs671 gene variant: A preliminary investigation with young East Asians

**DOI:** 10.1113/EP093300

**Published:** 2025-12-09

**Authors:** Beatrice Lioy, Wagner Ribeiro Pereira, Rehan Junejo, Tiago Peçanha, Guilherme Giannini Artioli

**Affiliations:** ^1^ Department of Life Sciences Manchester Metropolitan University Manchester UK; ^2^ Applied Physiology & Nutrition Research Group–Centre of Lifestyle Medicine, Faculty of Medicine, FMUSP University of Sao Paulo Sao Paulo Brazil; ^3^ LANEB–Laboratory of Nutritional & Exercise Biology, Department of Anatomy Institute of Biomedical Sciences University of Sao Paulo Sao Paulo Brazil; ^4^ Department of Sport and Exercise Sciences, Manchester Metropolitan University Institute of Sport Manchester Metropolitan University Manchester UK

**Keywords:** aldehyde dehydrogenase 2, aldehydes, endothelial function, flow‐mediated dilation

## Abstract

Aldehyde dehydrogenase 2 (ALDH2) is a mitochondrial enzyme that plays an important role in aldehyde detoxification. A large percentage (30–50%) of the East Asian population carry a single point mutation in the *ALDH2* gene (ALDH2*2 variant) that causes a severe reduction or lack of ALDH2 enzyme activity, and leads to disrupted cellular homeostasis due to the accumulation of toxic reactive aldehydes. The ALDH2*2 variant has been associated with several degenerative diseases, with evidence suggesting a link to cardiovascular disease, potentially mediated by endothelial dysfunction. This, however, remains to be confirmed. We aimed to investigate whether the ALDH2*2 variant is associated with impaired endothelial function in young, healthy East Asians. Twenty‐two participants were genotyped and divided into non‐carriers (ALDH2*1/*1; *n* = 12; 7 females and 5 males; age = 23 ± 3 years; height = 167.4 ± 8.7 cm; body mass = 60.1 ± 9.0 kg) and carriers (ALDH2*1/*2 and ALDH2*2/*2; *n* = 10; 8 females and 2 males; age = 24 ± 5 years; height = 162.6 ± 10.1 cm; body mass = 62.1 ± 9.7 kg) of the ALDH2*2 allele. Endothelial function was assessed via flow‐mediated dilation (FMD) following current guidelines. Carriers displayed lower FMD, either absolute or relative, which was not statistically significant but approached significance (unpaired *t*‐test) (FMD%: non‐carriers = 10.2 ± 1.9% vs. carriers = 8.1% ± 3.1%, *P *= 0.079, effect size: Cohen's *d* = 0.82; FMD_abs_: non‐carriers = 0.32 ± 0.06 mm vs. carriers = 0.26 ± 0.09 mm, *P *= 0.082, effect size: Cohen's *d* = 0.78). In conclusion, our data seem to suggest that the ALDH2*2 variant impairs endothelial function even in young and healthy individuals without the presence of other stressor agents. Future studies with larger sample size are necessary to confirm our findings.

## Abstract (highlights)

- **What is the central question of this study?**
  Do carriers of the mutation in the ALDH2 gene that results in sustained exposure to increased levels of reactive aldehydes display impaired endothelial function assessed with the flow‐mediated dilation (FMD) technique?
- **What is the main finding and its importance?**
  FMD was lower in young and healthy carriers of the mutated allele when compared to non‐carrier controls. The difference is physiologically relevant, but did not reach statistical significance. The findings are preliminary and suggest that sustained aldehydic load can have detrimental effects on vascular function, even in young and healthy individuals.

## 1 INTRODUCTION

Aldehyde dehydrogenase 2 (ALDH2) is a tetrameric mitochondrial enzyme encoded by the *ALDH2* nuclear gene (12q24). This enzyme plays an essential role in alcohol metabolism in liver cells and aldehyde detoxification in a range of tissues where it is expressed, such as the brain, the heart and skeletal muscle (Chen et al., 2014). Studies have shown that ALDH2 has cytoprotective effects against oxidative stress, lipid peroxidation, toxic aldehydes, and acute or chronic heart injury (Chen et al., 2008, 2014; Gomes et al., 2014, 2015; Ueta et al., 2018).

The *ALDH2* gene is the *locus* for the most common single nucleotide polymorphism known in humans. This dominant negative missense variant consists in a glutamate‐to‐lysine substitution at the position 504 of the peptide chain, and it is identified as ALDH2*2 (rs671). This allele is highly prevalent in East Asian populations, where ∼560 million individuals carry at least one copy, representing ∼8% of the world population. The ALDH2*2 variant causes diminished or absent ALDH2 catalytic activity, leading to the accumulation of aldehydes in several tissues and cells (Gross et al., 2015), and to a subsequent increase in oxidative and electrophilic stress (Singh et al., 2013). Since toxic aldehydes can damage biomolecules such as DNA and protein, aldehyde accumulation can cause a disruption in cell homeostasis and impair tissue function (Chen et al., 2014). The increased aldehydic load in carriers of the ALDH2*2 variant, even in heterozygosity, has been associated with increased risk for cardiovascular diseases (Guo et al., 2010; Li et al., 2017), including atherosclerosis (Cai et al., 2023).

Endothelium is a monolayer constituted by epithelial cells that envelopes the inner surface of several organs, including the blood vessels, where it serves as a regulator of blood flow, vascular tone and homeostasis (Esper et al., 2006; Guo et al., 2023). A large body of evidence establishes that endothelial dysfunction is a key factor involved in the aetiology of arterial hypertension, atherosclerosis and other cardiovascular diseases (Sitia et al., 2010). Endothelial function is influenced by a variety of genetic and environmental factors, and it can be negatively impacted by oxidative stress and disrupted redox balance. An increase in the production of reactive oxygen species (ROS) and products of lipid peroxidation such as cytotoxic aldehydes can lead to a pro‐inflammatory state and impair endothelial function and the ability of arterioles to dilate and regulate blood flow (Endemann & Schiffrin, 2004; Lynch et al., 2020). In fact, the deleterious effects of ROS and reactive aldehydes are not only detrimental to the endothelial function (Lynch et al., 2020), but have also been linked to pathogenic processes of numerous chronic degenerative diseases (Chen et al., 2014).

Guo et al. (2023) showed in a genome‐wide association study with data from Biobank Japan that the ALDH2*2 allele is one of the strongest single nucleotide variants associated with coronary artery disease. Whilst the association between ALDH2*2 and endothelial dysfunction‐related diseases suggests that the variant is a direct cause of endothelial dysfunction, the evidence to support this assertion remains limited to animal studies (Guo et al., 2023) or to studies with clinical cohorts that have already been exposed to other confounders such as ageing, smoking (Ma et al., 2015) and preexisting cardiovascular disease (Ma et al., 2015; Mizuno et al., 2020). To our knowledge, only one study investigated endothelium function in individuals with the ADLH2*2 variant (Guo et al., 2023). The authors used digital plethysmography to show impaired vasodilation in ALDH2*2 carriers compared to non‐carrier controls. This study, however, used a relatively small sample size and the participants were required to ingest alcohol before endothelial function assessment, limiting the understanding of whether endothelial dysfunction is constantly manifested, or only present under the influence of a strong stressor agent and a major source of aldehydes such as alcohol ingestion. Moreover, while digital plethysmography offers a convenient and non‐invasive means of assessing microvascular reactivity, it is generally considered less sensitive and less specific than FMD for detecting impairments in endothelial function and may provide less prognostic value for future cardiovascular risk (Babcock et al., 2020; Dirjayanto et al., 2024; Wilk et al., 2013).

Here, we hypothesised that the ALDH2*2 variant is positively associated with endothelial dysfunction in humans; to test this and to further understand whether endothelial dysfunction can be manifested irrespective of additional stressor agents, we examined endothelial function in young, healthy East Asians by means of the flow‐mediated dilation (FMD) method.

## 2 METHODS

### 2.1 Ethical approval

This study was conducted according to the principles in the *Declaration of Helsinki*, except for the registration in a database. All procedures were approved by the Faculty of Science and Engineering Research Ethics Committee of the Manchester Metropolitan University (Ref. No. FREGSE_UG/PGT_022_02.11.2022). Each participant provided written consent after being fully informed of the purpose of the study, the experimental procedures, and all potential risks and discomforts related with their participation. Recruitment and data collection were carried out in the Institute of Sport (Manchester Metropolitan University, Manchester, UK) between December 2022 and August 2023.

### 2.2 Study design and participants

This was a cross‐sectional study to determine the association between the *ALDH2* gene mutation and endothelial function assessed by means of FMD. Healthy East Asian participants (i.e., with Chinese, Japanese and Korean ancestry) aged between 18 and 40 were eligible to take part in this study. All participants were residing in Manchester at the time of data collection. Exclusion criteria were: smoking or vaping (current or past), presence of any infectious diseases in the past 2 weeks, diagnosis of any conditions or disorders related to endothelial function, or the use of any medications that could affect endothelial function. Participants were recruited through direct oral communication or via social media, fliers and posters on the university campus. Twenty‐nine individuals responded to the invitations and were assessed for eligibility, of which three were ineligible. Eligibility assessment involved an online conversation where the participants completed a medical screening questionnaire, which included questions on the frequency and the amount of alcohol intake. Twenty‐six participants attended the lab for data collection, but four had their data lost due to a malfunction in the data recording system, with full data being available for 22 participants (Chinese: *n* = 20; Korean: *n* = 1; Japanese: *n* = 1; female: *n* = 15; male: *n* = 7).

Endothelial function was measured in all participants via FMD. They were later genotyped for the *ALDH2* gene mutation and the researchers kept blind to the genotypes during data collection and analysis. After all FMD data were extracted, participants were divided into two groups according to their genotypes: non‐carriers (ALDH2*1/*1, *n* = 12) and carriers (ALDH2*1/*2, *n* = 7 and ALDH2*2/*2, *n* = 3).

### 2.3 Data collection and FMD procedures

Participants were required to attend to the laboratory on one occasion for a mouthwash collection and FMD analysis. The visits were scheduled to take place in the morning to ensure they would adhere to an overnight fast and to hydration recommendations. This also ensures minimal interference of circadian factors on FMD data. They were instructed to refrain from alcohol, physical exercise and caffeine in the 24 h prior to their visit, and to avoid food and drinks, except water, for the 8 h before the visit. They were also instructed to come well‐hydrated for data collection, but without having ingested water 2 h before the tests. Compliance with these requests were verbally confirmed.

Upon arrival, all participants completed the 7‐item short version of the International Physical Activity Questionnaire (IPAQ) to assess physical activity levels, and the alcohol intake questionnaire. They then undertook a vigorous mouthwash with saline solution for buccal mucosa cell collection, and were asked to lie quietly in the supine position on an examination bed and relax for 10 min. The collection room was kept silent and free of noise throughout data collection. Blood pressure and heart rate measurements were taken three times with 45 s intervals using a digital blood pressure monitor (OMRON M2, Omron Healthcare, Milton Keynes, UK).

FMD was evaluated according to current guidelines (Thijssen et al., 2019) using a high‐resolution ultrasound machine (Terason uSmart 3300, Terason, Burlington, MA, USA) equipped with a 4.0–15.0 MHz linear transducer. Initially, participants lay down in the supine position with their arm extended at an angle of ∼80° from the torso. A pneumatic cuff was positioned at the participants’ forearm to provide an ischaemic stimulus. Longitudinal images of the brachial artery diameter were taken using B‐mode ultrasound, and simultaneous pulse‐waved Doppler blood flow velocity was obtained using a <60° insonation angle, with the sample volume placed in mid‐artery and aligned with the blood flow. Initially, a 1‐min baseline recording of the brachial artery diameter and blood flow velocity was performed and then the forearm cuff was inflated (∼200 mmHg) for 5 min. Recordings were resumed 30 s before cuff deflation and continued for 3 min thereafter. Brachial artery diameter and shear rate (4 × mean blood velocity/internal diameter) were analysed by a blinded evaluator using semi‐automatic edge‐detection and wall‐tracking software (Cardiovascular Suite, Quipu®, Pisa, Italy). FMD% was calculated as the percentage change of the vessel diameter after cuff release in relation to baseline vessel diameter (FMD = [(*D*_max_ − *D*_baseline_)/*D*_baseline_] × 100). Baseline and peak diameters were log‐transformed to determine regression slopes and upper bound 95% confidence intervals. As the slopes were consistently above 0.9 and upper bound confidence intervals above 1.0, allometric scaling for baseline diameters was not required (Atkinson & Batterham, 2013). To describe the relevant shear rate stimulus for FMD, we also calculated the area‐under‐the‐curve of the shear rate up to the peak diameter (SRAUC). Other parameters obtained from the FMD evaluation, including brachial artery baseline diameter, peak diameter, absolute dilation (FMD_abs_) and time‐to‐peak diameter were also calculated and reported (Thijssen et al., 2019).

### 2.4 DNA isolation and extraction

Genomic DNA was isolated from buccal epithelial cells obtained from mouthwashes. Approximately 30 mL of saline solution (0.9% NaCl) prepared in nuclease‐free water was given to the participants in a sterile 50 mL tube; they were asked to vigorously rinse their mouth for 30 s and spit the wash back to the tube. The samples were stored at −20°C until analysis. After thawing, the samples were centrifuged at 8000 *g* for 5 min and the supernatant was dispensed. The pellets containing buccal cells were resuspended and incubated overnight with 700 µL of lysis buffer (Tris–HCl 10 mM, pH 8.0, EDTA 100 mM, pH 8.0, NaCl 100 mM, SDS 0.5%, Proteinase K 100 µg/mL) under agitation at 55°C. Chloroform (400 µL) was added and the samples centrifuged at 5000 *g* for 5 min. The upper layer was carefully transferred to a clean microtube and DNA was precipitated by adding 1 mL of cold absolute ethanol. DNA was pelleted by centrifuging at 5000 *g* for 5 min. The supernatant was dispensed, and tubes were left inverted for 15 min to allow alcohol evaporation. Nuclease‐free Tris‐EDTA (TE) buffer was used to resuspend the isolated DNA. DNA quality and concentration were assessed in a microspectrophotometer (NanoDrop One, Thermo Fisher Scientific, Waltham, MA, USA).

### 2.5 Genotyping and PCR analysis

One microliter of the DNA was mixed with 10 µL of the Genotyping PCR Master Mix (TaqMan Genotyping Master Mix®, Thermo Fisher Scientific), 1 µL of the TaqMan VIC/FAM probe and primer specific for the *ALDH2* rs671 polymorphism genotyping (TaqMan® SNP Genotyping Assays, Thermo Fisher Scientific), and the final volume completed to 22 µL. The reactions conditions were as follow: initial hold temperature at 95°C for 10 min; 40 cycles of denaturation at 92°C for 15 s and annealing/extension (two‐step cycling protocol) at 60°C for 60 s.

### 2.6 Statistical analysis

Student's unpaired *t*‐test was used to compare the baseline characteristics and all FMD parameters between non‐carriers and carriers. Upon data inspection, we noted a few extreme values, and so an outlier detection analysis was conducted (Grubbs’ test, α = 0.2). The analysis confirmed the presence of one outlier on each group for %FMD, and one outlier for absolute FMD in the carriers group. These data points were then removed. An a priori sample size estimation was calculated using an online application (Dhand & Khatkar, 2014) considering a 4% between‐genotype difference in relative FMD (a plausible size of effect for a factor that moderately affects endothelial function), 3% standard deviation, 1 − β = 0.8, and α = 0.05 for a two‐sided unpaired *t*‐test. According to this estimation, a total sample of 24 participants would be required (*n* = 12 in each group); this means that our final analysis is likely to be slightly underpowered. Alcohol frequency as well as sex distribution was compared between genotype groups with Fisher's exact test. Effect sizes were calculated for absolute and relative FMD using the Cohen's *d* equation and interpreted as small if 0.2 > *d* < 0.5, medium if 0.5 > *d* < 0.8, and large if *d* > 0.8. All values are expressed as means ± standard deviation (SD), and the statistical tests were carried out using Prism 8 software (GraphPad Software, San Diego, CA, USA).

## 3 RESULTS

Participants baseline characteristics were not significantly different between groups (age, height, body weight, systolic blood pressure, diastolic blood pressure, heart rate, baseline diameter, peak diameter and time to peak diameter of brachial artery; all *P *> 0.05, Table 1).

**FIGURE 1:**
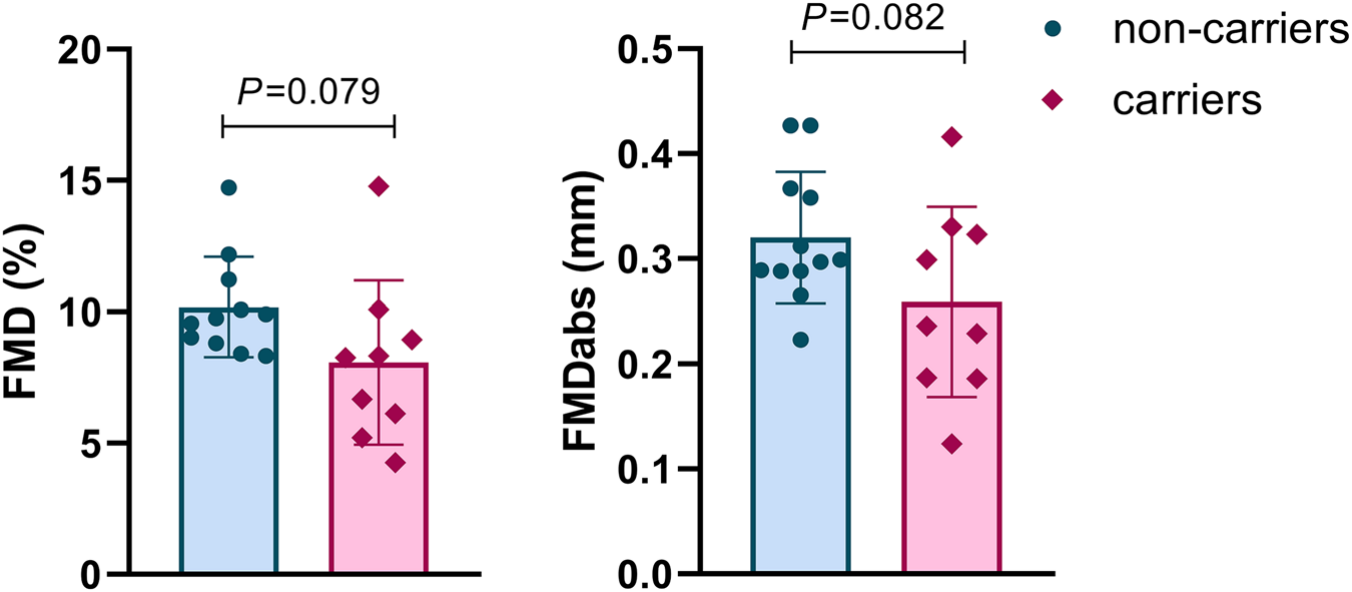
Absolute and relative flow‐mediated dilation in carriers and non‐carriers of the rs671 variant of the *ALDH2* gene.

FMD values, either absolute or relative were lower in the ALDH2*2 group and marginally different compared with the non‐carriers (FMD%: *t *= 1.859, *P *= 0.079; Cohen's *d* = 0.82, large; FMD_abs_: *t *= 1.833, *P *= 0.082; Cohen's *d* = 0.78, medium; Figure 1).

**TABLE 1:** Baseline characteristics of the participants.

	Non‐carriers (ALDH2*1/*1)	Carriers (ALDH2*1/*2 and ALDH2*2/*2)	*P*
Female (*n*)	7	7	—
Male (*n*)	4	2	0.642
Age (years)	23 ± 3	24 ± 5	0.674
Height (cm)	167.4 ± 8.7	162.6 ± 10.1	0.244
Body weight (kg)	60.1 ± 9.0	62.1 ± 9.7	0.633
BMI (kg/m^2^)	21.4 ± 2.0	23.4 ± 2.7	0.080
Systolic blood pressure (mmHg)	111.1 ± 12.0	108.9 ± 9.5	0.645
Diastolic blood pressure (mmHg)	72.4 ± 7.7	73.6 ± 9.9	0.746
Heart rate (bpm)	66.4 ± 12.4	61.9 ± 7.8	0.334
Baseline diameter (mm)	3.2 ± 0.4	3.2 ± 0.6	0.907
Peak diameter (mm)	3.6 ± 0.2	3.5 ± 0.6	0.418
Time to peak diameter (s)	85.2 ± 61.0	52.1 ± 14.0	0.129
Physical activity score (MET‐min/week)	2138 ± 1089	2994 ± 2226	0.436
Alcohol frequency (times/week)	1.75 ± 0.97	2.00 ± 0.82	0.189
Alcohol intake (units/week)	1.50 ± 0.80	1.30 ± 0.48	0.646

## 4 DISCUSSION

To our knowledge, this is the first study to evaluate endothelial function using a direct method in young, healthy carriers of the *ALDH2* human gene mutation. We hypothesized that the mutated allele, whether in homozygosity or heterozygosity, would be linked to endothelial dysfunction as a consequence of sustained accumulation of reactive aldehydes and their deleterious effects on the endothelium. Previous literature has shown that increased aldehydic load that accompanies low ALDH2 activity leads to cytotoxicity and disrupted homeostasis (Chen et al., 2020, 2014; Pereira et al., 2023), with some authors (Ma et al., 2015) attributing to the ALDH2*2 variant a link between aldehyde accumulation and endothelial dysfunction. Here, we provided preliminary evidence that the ALDH2*2 variant associates with impaired endothelial function. Whilst our results are not statistically significant, they approached significance and the effects sizes are moderate‐to‐large; if further confirmed, this could indicate that the mutation might be a factor leading to reduced endothelial function.

Previous studies have already suggested that the *ALDH2* gene mutation is a major genetic risk factor for coronary artery disease (Guo et al., 2010; Li et al., 2017; Xu et al., 2011; Zhang et al., 2023). This has been confirmed in a mice Aldh2*2 knock‐in model mimicking the human heterozygous ALDH2*1/*2 genotype (Guo et al., 2023), and in humans with pre‐existing cardiovascular disease (Ma et al., 2015).

The present study reinforces the concept that *ALDH2* mutation can affect endothelial function in humans, and further adds the notion that this can be manifested at very early stages, even in young healthy individuals without any additional stressor agent or exogenous source of aldehydes. These detrimental effects could potentially become more pronounced during ageing and interact with age‐related pathological processes, as the toxic effects of aldehyde can accumulate over time. Moreover, they could be further aggravated in conditions where lipid peroxidation is more pronounced (e.g., smoking, alcohol intake, or some chronic disease). Since the differences in FMD between genotypes were not statistically significant in our study, future studies could confirm these findings and expand them to older populations, or by examining whether behavioural and environmental factors, such as smoking and exposure to pollution could aggravate these effects.

In line with our findings, the study by Ma et al. (2015) demonstrated that the ALDH2*2 allele is over‐represented in hypertensive patients with compromised FMD% compared to healthy controls. The authors concluded that the ALDH2*2 allele is associated with hypertension, which is a condition where endothelial dysfunction is most certainly present. Importantly, Ma et al. (2015) did not exclude smokers and older individuals from their sample, which could be a confounding factor in assessing whether the ALDH2*2 variant is implicated with hypertension and endothelial dysfunction. More recently, Guo et al. (2023) showed impaired vasodilation in ALDH2*2 participants compared to ALDH2*1 following alcohol ingestion. Our study, therefore, further indicates that ALDH2*2 per se already represents a burden to endothelial function and vascular homeostasis.

Although our results align well with the current knowledge, and despite the study being designed to refine previous investigations with the use of better experimental controls, more direct assessment of endothelial function, and a cohort with less confounding factors, we acknowledge that our study has some important limitations that need to be highlighted. This is a small‐scale study with limited sample size; the statistical power was, therefore, insufficient to detect significant differences in FMD between genotypes, although these differences were physiologically relevant, and of moderate‐to‐large effect sizes. Nonetheless, the level of certainty of these findings should be weighed and further studies confirming the results are needed. Additionally, we did not control for habitual alcohol intake or physical activity during recruitment. Although these are two major factors that can modulate vasodilation, adding them to the exclusion criteria could result in an excessively stringent criteria and make participant recruitment impractical. To account for this, we collected self‐reported information on alcohol intake and physical activity; these were not different between genotypes, thereby alleviating the importance of this limitation. Moreover, whilst FMD is a gold‐standard method for the non‐invasive measurement of endothelial function in humans and an early marker of atherosclerosis and future cardiovascular events (Daniele et al., 2024; Stoner et al., 2013), it also has important limitations, such as the influence of baseline artery diameter on the results and the inter‐operator. To minimise measurement error in our study, all procedures were undertaken by a single trained operator following current guidelines (Thijssen et al., 2019). Finally, genotype–phenotype association studies have inherent limitations, including the poor control of unknown confounding variables, which can be circumvented with the use of large sample sizes; this highlights that the low sample size must be taken into account when interpreting our findings.

To conclude, our study provided preliminary evidence for reduced endothelial function as a consequence of carrying the ALDH2*2 variant in young and healthy individuals. This highlights the importance of early screening for chronic diseases in these populations. Future studies should confirm these findings and explore whether mitigation strategies (e.g., exercise and nutrition) could rescue normal function.

## AUTHOR CONTRIBUTIONS

Guilherme Giannini Artioli conceived the study. Guilherme Giannini Artioli, Tiago Peçanha, and Rehan Junejo designed the research. Beatrice Lioy, Rehan Junejo, and Tiago Peçanha conducted the experiments. Beatrice Lioy and Guilherme Giannini Artioli analysed the samples. All authors analysed and interpreted the data. Wagner Ribeiro Pereira and Guilherme Giannini Artioli drafted the manuscript and prepared the figures. Wagner Ribeiro Pereira, Tiago Peçanha, and Guilherme Giannini Artioli edited and revised the manuscript. All authors approved the results of the study and the final version of the manuscript and agree to be accountable for all aspects of the work in ensuring that questions related to the accuracy or integrity of any part of the work are appropriately investigated and resolved. All persons designated as authors qualify for authorship, and all those who qualify for authorship are listed.

## CONFLICT OF INTEREST

None declared.

## Data Availability

Data presented in this manuscript is available by contacting the corresponding author.
